# How precise is the identification of the center of the femoral head during total knee arthroplasty?

**DOI:** 10.3109/17453674.2011.641106

**Published:** 2012-02-08

**Authors:** Jai-Gon Seo, Young-Wan Moon, Sang-Hoon Park, Ho-Min Kang, Sang-Min Kim

**Affiliations:** Department of Orthopedic Surgery, Samsung Medical Center, Sungkyunkwan University School of Medicine, Seoul, Korea

## Abstract

**Background and purpose:**

Identification of the center of the femoral head in the coronal plane is essential during total knee arthroplasty. We evaluated a new method for localization of the center of the hip, thereby detecting the neutral mechanical axis using inter-femoral head center distances (X) measured from a radiograph. Our proposed method was compared with 3 commonly used methods using landmarks that are estimated to be 2 finger-breaths medial to the anterosuperior iliac spine (method A), 2.5 cm perpendicular to the mid-inguinal point (method I), and 1.5 cm lateral to the femoral artery (method F).

**Methods:**

114 patients undergoing total knee arthroplasty were prospectively enrolled in the study. Four landmarks were marked and conventional anterior-posterior pelvic radiographs were taken. On the radiograph, the distance between the estimated FHC and the neutral mechanical axis was measured.

**Results:**

The median value (mm) of the measured distance was 9 in A, 7 in I, 8.5 in F, and 5 in X. When an error of more than 3° from neutral alignment was defined as an outlier, 15% of measurements in A, 6% of measurements in I, 14% in F, and 2% in X would fall in the outlier zone.

**Interpretation:**

The method detecting the neutral mechanical axis using inter-femoral head center distances (X) showed the least variability and the lowest percentage of outliers.

Correct alignment is important for the longevity of total knee arthroplasty (TKA) (Jeffery et al. 1991, Ritter et al. 1994, Ensini et al. 2007, Sikorski 2008). Location of the center of the femoral head (FHC) intraoperatively is useful in assessment of the overall alignment of the lower limb during TKA. By estimating the mechanical axis after placement of the trial components, errors of limb alignment can be identified and corrected.

Ideally, the FHC can be identified by an on-table radiograph, which, however, is time consuming and inconvenient and exposes the patient to additional radiation. Navigation systems have become more widely used to find the FHC and they may improve the accuracy of alignment, but this approach is not always available. Palpation of the anterior iliac spine (ASIS) is commonly used intraoperatively to indirectly estimate the center of the femoral head (Horton and Reckling 1995, Hooper et al. 2005). However, some authors have suggested that this is not as accurate as it is commonly presumed to be (Mullaji et al. 2010, Baldini and Adravanti 2008). Various methods using anatomical landmarks have been reported as alternatives (Horton and Reckling 1995, Matsuda et al. 2004, Sawant et al. 2004, Samarji et al. 2009). They include the use of a landmark that is located 2 finger-breaths medial to the ASIS (method A) (Hooper et al. 2005), a landmark that is located 1.5 cm lateral to the point where the femoral artery crosses the line joining the pubic tubercle and the ASIS (method F) (Sawant et al. 2004), and a landmark that is located 2.5 cm perpendicular to the midpoint of the line joining the ASIS and the symphysis pubis (method I) (Samarji et al. 2009).

Here we describe a new method (X) for localization of the FHC using the inter-femoral head center distance (IFD). The IFD was measured on an anteroposterior radiograph of the pelvis preoperatively. A customized metal graduated ruler with 2 mobile pegs was used to replicate the IFD, and this ruler was fitted above the pelvic girth. Thus, these 2 pegs indicated the FHC and then we could identify the neutral mechanical axis of the lower limb.

In this study, we validated the reliability of method I in identifying the neutral mechanical axis of the lower limb in vivo, and compared the precision of the methods using A and X. We also evaluated the 2 techniques using F and I. In addition, we tried to determine whether height, body mass index (BMI), and abdominal circumference had any influence on the 4 methods.

## Materials and methods

### Study subjects

Before starting the study, we obtained the approval of the institutional review board. 114 patients (107 women) undergoing primary unilateral TKA were prospectively enrolled between August 2010 and October 2010 after first obtaining their informed consent. We excluded those subjects who had a history of (1) trauma or surgery to the pelvis or hip, (2) neuromuscular disorders, (3) hip deformity or limp, or (4) limb deformity at any level.

The mean age of the patients was 68 (43–85) years. The mean height was 153 (137–190) cm, with 58 subjects less than 153 cm and 56 subjects equal to or more than 153 cm. The mean BMI was 27 (20–35), with 61 subjects below 27 and 53 subjects equal to or above 27. The mean abdominal circumference (AC) was 92 (75–132) cm with 57 subjects measuring less than 90 cm and 57 subjects measuring 90 cm or more.

**Figure 1.**
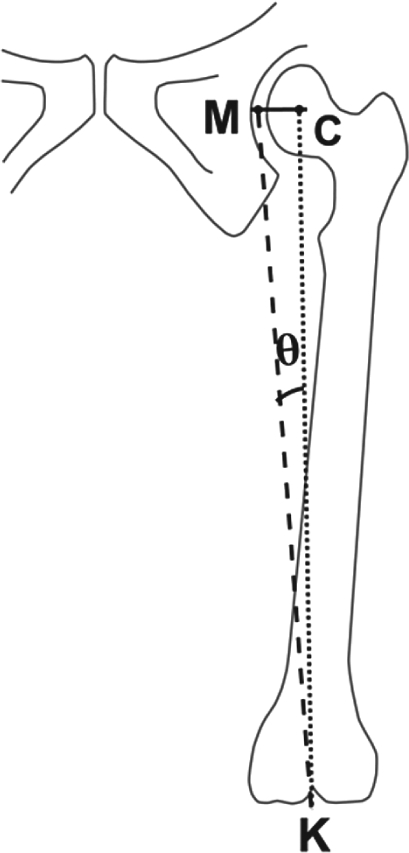
Coronal alignment error calculated trigonometrically.

Standing AC (cm) at umbilical level was also measured for each patient. From the mean femoral length of 376 mm in our population, the angular errors that might occur in the coronal plane were calculated trigonometrically (Sawant et al. 2004) (Figure 1). We compared data from those with the BMI of more than or less than 27, the AC of more than or less than 90 cm, and subjects with standing height of more than or less than 155 cm.

M: estimated center of head; C: true center of head; K: center of knee; θ: angular alignment error.

### Determination of 4 landmarks

**Figure 2.**
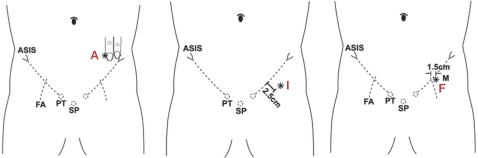
Illustration of 3 landmarks for determination of the center of the femoral head: A (left panel); I (center panel); F (right panel). ASIS: anterosuperior iliac spine; FA: femoral artery; PT: pubic tubercle; SP: symphysis pubis.

**Figure 3.**
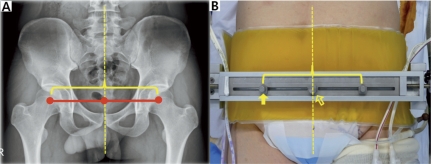
A. The distance between femoral head centers on both sides was measured on a radiograph of the pelvis. B. A customized metal ruler with two mobile pegs was developed to replicate the inter-femoral head distance. Dotted line: pelvic midline; empty arrow: center rod; solid arrow: mobile peg (landmark X).

The subjects were kept in a supine position on the operation table, with hip and knee in full extension and patellae facing upwards before surgery. Afterwards, the positions of 3 existing landmarks (A, I, and F) were identified based on the methods described in previous studies (Sawant et al. 2004, Hooper et al. 2005, Samarji et al. 2009) (Figure 2). With regard to the method using F, portable doppler sonography was used only if the femoral artery could not be palpated easily. The location of the currently proposed landmark (X) was determined as follows. First, the distance between hip centers on both sides was measured on anteroposterior radiographs of the pelvis using the Picture Archiving and Communication System (PACS; General Electric, Milwaukee, WI). To find the degree of magnification, all radiographs were taken with a 10-cm-sized metal bar attached to the skin of the proximal thigh of a patient. The degree of magnification was calculated using the 10-cm bar and the measure accordingly corrected (Seo et al. 2010). This value was then defined as the IFD. With the patient supine, a customized graduated ruler with 2 mobile pegs was fitted to the assembly-type metal pelvic stabilizer that could contain the pelvis of a patient. The distance between the 2 mobile pegs on the ruler replicated the IFD (Figure 3). The center rod of this ruler was located on the pelvic midline passing through the symphysis pubis and xiphoid process, which were easily palpated through the surgical drapes. Assuming that pelvic structures were bilaterally symmetrical and that the IFD was perpendicularly bisected by the pelvic midline, the distances between the 2 pegs were bisected by the center rod. Thus, these pegs indicated the estimated centers of the femoral heads (landmark X).

### Measurement of errors in estimation of the neutral mechanical axis

4 landmarks were marked with adhesive ECG electrodes with peripheral rim trimmed. The electrode is radio-opaque and has a prominent metallic center. A conventional anterior-posterior radiograph of the pelvis was taken to confirm the location of these markers. On the radiograph, the positional error in the estimation of the neutral mechanical axis of the lower limb was measured. The neutral mechanical axis of the lower limb passing the real center of the femoral head was drawn using a template of concentric circles. The other vertical line passing the center of each landmark was also drawn. The horizontal distance between the 2 vertical lines was measured to the closest mm, and adjusted for magnification. The location of each landmark relative to the neutral mechanical axis was also recorded. It was classified into 3 areas: M, medial; L, lateral; and C, center area within 2 mm of the horizontal distance from the real FHC. To investigate inter- and intraobserver variability, 4 landmark positions were measured by 2 independent observers, and twice on different occasions by 1 observer in all cases.

### Statistics

The sample size was determined based on values that were derived from the pilot study involving 20 patients. With mean values of distance between the real and estimated FHC of 6.0 mm (SD 2.7) for the X method and of 8.2 mm (SD 6.7) for the A method, we determined that 114 cases would be needed to show a statistically significant difference with a power (1 – β) of 80% (α = 0.05). The descriptive statistics for continuous variables are reported as median, interquartile range, and 95% confidence intervals (CIs). For the comparisons between the four methods, continuous variables were analyzed using a mixed model after square-root transformation of the variables that were not normally distributed. Categorical variables were analyzed using Wilcoxon 2-sample test. We used Tukey's test for multiple testing. Bonferroni's method was used to correct the inflated type-I error due to multiple testing. All the analyses were performed using SAS software version 9.1 (SAS Institute, Cary, NC) with statistical significance set at p-values of less than 0.05. Intraclass correlation coefficients (ICCs) were used to determine the degree of agreement for one rater or between raters

## Results

The relative precision differed between the 4 methods (mixed model, p < 0.001) (Table 1). The most precise technique appeared to be the X method, compared to the A, the I, and the F methods (Tukey's test for multiple testing, p < 0.001, p = 0.003, and p < 0.001, respectively). The I method was more accurate than the A and the F methods (Tukey test for multiple testing, p = 0.003 and p = 0.02, respectively). There was no statistically significant difference in accuracy between the A and the F methods.

**Table 1.** Distances (mm) between the estimated and real femoral head centers

Method				
	A	I	F	X
Median (Q1, Q3)	9 (6, 15)	7 (4, 12)	8.5 (5, 15)	5 (3, 7)
Range	0–25	0–22	0–28	0–18
98.75% CI **^a^**	8–12	6–9	7–12	4–6

**^a^**98.75% CI was corrected due to multiple testing; Q1=25th percentile; Q3=75th percentile; CI=confidence interval.

**Figure 4.**
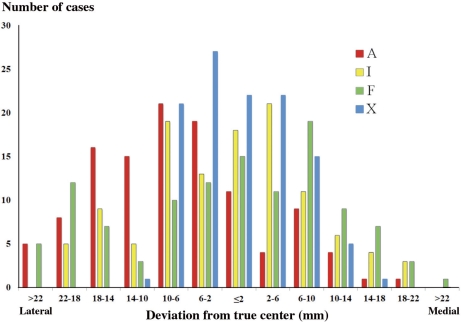
Overall distribution for each method, indicating the number of cases at intervals of 4 mm.

Each landmark of the above 4 methods was found in the 3 areas (area M, C, and L) with reference to the neutral mechanical axis of the lower limb (Table 2). The A landmark had a strong tendency to be located on the area L, unlike the other methods (Table 2 and Figure 4).

**Table 2.** Location of each landmark of the 4 methods

Methods	Area M	Area C	Area L
A	19 (17%)	11 (10%)	84 (74%)
I	45 (39%)	19 (17%)	50 (44%)
F	50 (44%)	15 (13%)	49 (43%)
X	45 (39%)	19 (17%)	50 (44%)

M, medial; L, lateral; C, center area (±2 mm)

Using distances between the estimated and real FHC, trigonometric analysis was performed to calculate the angular errors in coronal alignment that would result if these values were used as reference. For 6.6 mm, 13 mm, and 20 mm differences in the coronal plane, the angular change (θ) was 1°, 2°, and 3°, respectively. If a surgical outlier was defined as a deviation greater than 3°, the X method showed the lowest number of surgical outliers (2/114, 2%) while the A method showed the largest number of outliers (17/114, 15%) (Table 3). The measured errors of each method were compared between the 2 groups, which were dichotomized by BMI, height, and AC (Table 4). In the A and the F method, the distance between the estimated and real FHC differed significantly between the subjects with a BMI of less than 30 and a BMI of 30 or more (Wilcoxon 2-sample test, p = 0.01 and p = 0.01, respectively). Also, there was a significant difference between the subjects with an AC of less than 95 cm and those with an AC of 95 cm or more in the A method (Wilcoxon two-sample test, p = 0.01).

**Table 3.** Amount of errors in the estimation of the real femoral head center. Values are number of cases (percentage) within limit.

Method	Distances between the estimated and real femoral head centers (mm) (angular error, θ)		
	≤ 6.6 (≤ 1°)	≤ 13.1 (≤ 2°)	≤ 19.7 (≤ 3°)
A	34 (30%)	73 (64%)	97 (85%)
I	52 (46%)	92 (81%)	107 (94%)
F	39 (34%)	76 (67%)	98 (86%)
X	83 (73%)	106 (93%)	112 (98%)

**Table 4.** Factors affecting results of the four methods. Values are median distance (mm) and (Q1, Q3)

Method	Height (cm)		Difference **^a^**	BMI (kg/m²)		Difference **^a^**	AC (cm)		Difference **^a^**
	< 153	≥ 153		< 27	≥ 27		< 90	≥ 90	
A	11 (9, 18)	9 (6, 14)	0.29	9 (6, 12)	14 (6, 18)	0.01	9 (6, 10)	14 (8, 18)	0.01
I	7 (4, 13)	7 (3, 10.5)	0.8	7 (4, 11)	7 (4, 12)	1.0	7 (3, 11)	7 (4, 12)	0.9
F	12 (6, 18)	7 (3.5, 12.5)	0.06	7 (3, 12)	13 (8, 20)	0.01	7 (4, 13)	12 (6, 18)	0.67
X	5 (3, 7)	5 (3, 7.5)	1.0	5 (3, 7)	5 (3, 8)	0.88	5 (3, 7)	5 (3, 8)	0.07

**^a^**Wilcoxon two-sample test; BMI=body mass index; AC=abdominal circumference

The ICC for intraobserver variability was 0.68 for A, 0.70 for M, 0.6 for F, and 0.83 for X. The ICC for interobserver variability was 0.63 for A, 0.64 for I, 0.5 for F, and 0.76 for X. Thus, there was good or excellent intra- and interobserver agreement in all of the measurements performed.

## Discussion

In TKA, ideally the postoperative coronal alignment of the lower limb should be a straight line passing through the center of the hip, the center of the knee, and the center of the ankle. We wanted to identify the degree of error in finding the FHC and thereby detect the neutral mechanical axis of the lower limb.

Previous studies have suggested the use of ASIS as an important bony landmark from which to determine the location of the FHC (Ritter and Campbell 1988, Cates et al. 1993, Seidel et al. 1995, Hooper et al. 2005). However, TKA using only ASIS as an intraoperative landmark for the referencing instruments has led to inferior radiographic outcome (Engh and Petersen 1990, Cates et al. 1993, Laskin 2001, Baldini and Adravanti 2008). In the present study, there was a wide variation in error, and the largest amounts of outliers in the ASIS method. Also, this method was affected by BMI and AC.

As a possible alternative to the ASIS method, some authors have described the use of vascular landmarks, and ultrasound to identify the center of the femoral head. Horton and Reckling (1995) measured the coronal plane distance between the femoral artery 2.5 cm below the inguinal ligament and the center of the femoral head on a pelvic arteriogram. Using this method, it was found that the femoral artery was within 15 mm of the FHC in 93% of cases. Matsuda et al. (2004) showed that the ultrasound identified FHC within 10 mm in 90% of cases. We found, using femoral arterial pulse, that the distance between the estimated FHC and the real FHC was within 20 mm (angular error ≤ 3°) in 85% of patients, which indicates that there was a larger proportion of outliers compared to the above studies using vascular landmarks with or without ultrasound. Also, the error in distance between the estimated and real FHC was substantial in patients with obesity. Samarji et al. (2009) proposed a palpable marker placed 2.5 cm perpendicular to the mid-inguinal point for detection of the FHC. They reported that the mean horizontal distance between the marker and a vertical line passing through the FHC was 8.6 mm, and was not related to AC. These results are comparable to our results.

Our new method for localizing the FHC using IFD and thereby identifying the neutral mechanical axis of the lower limb used radiographic measurements as reference. Though Mullaji et al. (2010) reported that the IFD of an Indian population showed a narrow range in 75% of individuals—of 16 (± 1) cm—we judged it inappropriate to assume the same value of IFD in our patients. Thus, the IFDs of all patients were measured preoperatively. Using this method, the accurate distance from pelvic midline to the FHC could be obtained and a newly developed graduated ruler was used to replicate this distance. This customized ruler has been included in older systems, but with fluoroscopic positioning (Hood et al. 1981, Tillett et al. 1988, Engh and Petersen 1990). Through these procedures, the limitation that the FHC is indirectly determined related with anatomical structures adjacent to the femoral head was thought to be overcome. When 3 anatomical methods (A, I, and F) and our proposed method (X) were studied at the same time, a statistically significant difference was found in the distance between the estimated and real FHC. The X method was the most consistent and precise one. If the theoretical mechanical axis model is used, our method would achieve the desired coronal alignment with an accuracy of equal to or less than 3° in 98% of cases.

There are some concerns about our proposed method. First, errors occur when applying the ruler. To determine the position of the IFD, we presumed that the structure of the pelvis is bilaterally symmetrical and that the IFD is perpendicularly bisected by the pelvic midline. If the IFD is not bisected by the pelvic midline, the distance between the 2 pegs indicating the IFD is not evenly divided. In practice, we readjust the ruler when there is substantial asymmetry because of previous pelvic surgery, trauma, or deformity—although such cases were excluded in this study. Also, the inappropriate placement of the ruler over the midline may be a source of error. To evaluate the potential error caused by these possibilities, the distance between symphysis pubis and the hip centers on both sides was investigated in all cases. The average difference for both sides was 2.3 mm (SD 1.4). When this value was converted to the angular change by trigonometric calculation (Sawant et al. 2004) with a femoral length of 376 mm, the angular error was 0.4° (SD 0.2) in the coronal plane. We believe that this error will not influence the accuracy of the X method. Secondly, the metal peg representing the IFD might be dislodged during surgery. This problem was resolved by developing the assembly-type metal pelvic stabilizer, which could contain the pelvis of a patient. The graduated ruler was firmly fitted to this pelvic stabilizer and the position of the IFD was not altered during the surgical procedure. Thirdly, another question is whether our method is easy to use, especially in the presence of a sterile draping in the operating field. We believe that our results could form the basis of development of a more accurate and consistent method than the ones currently available.
